# Three Cases of Acute Necrotizing Encephalopathy: Is It an Epidemic or Only Incidental?

**Published:** 2020

**Authors:** Farzad AHMADABADI, Sonia RUHOLLAHI, Reza MASKANI, Narjes JAFARI, Sanaz KARIMI DARDASHTI

**Affiliations:** 1 Department of Pediatrics Neurology, Ardabil University of Medical Sciences (ARUMS), Ardabil, Iran; 2 Radiologist, Ardabil University of Medical Sciences (ARUMS), Ardabil, Iran; 4 Department of Pediatrics Neurology, Shahid Beheshti University of Medical Sciences (SBMU), Tehran, Iran; 5 General Surgeon, Ardabil University of Medical Sciences (ARUMS), Ardabil, Iran

**Keywords:** Acute necrotizing encephalopathy (ANE), Seizure, Pediatric

## Abstract

Acute necrotizing encephalopathy of childhood (ANEC) is a disease, characterized by a respiratory or gastrointestinal infection, accompanied with fever, rapid alteration of consciousness, and seizures. The clinical characteristics of ANEC include acute encephalopathy following a viral infection, seizure, altered consciousness, and absence of cerebrospinal fluid (CSF) pleocytosis, with an occasional increase in the level of proteins. This disease is almost exclusively seen in previously healthy infants and children from East Asia. Serial magnetic resonance imaging (MRI) examinations have demonstrated symmetric lesions involving the thalami, brainstem, cerebellum, and white matter. ANEC has a poor prognosis with high morbidity and mortality rates. Herein, we present three cases of ANEC, who were referred to Bu-Ali Hospital of Ardabil, Iran during two weeks. Report of these three cases promoted the idea of an epidemic. The purpose of this case series was to raise the issue that ANEC may occur as an epidemic.

## Introduction

Acute necrotizing encephalopathy of childhood (ANEC) or acute necrotizing encephalopathy (ANE) is an atypical encephalopathy, which is almost exclusively seen in previously healthy young children and infants from East Asian countries, including Japan and Taiwan (1). Nevertheless, the sporadic incidence of this disease has been reported all around the world (2, 3). Although the etiology and pathogenesis of ANEC remain unclear, mycoplasma, influenza virus, herpes simplex virus (HSV), and human herpesvirus-6 are among the most common infections that intensify this disease (4-5). 

It is believed that ANEC is similar to metabolic and immune-mediated reactions (6). It initiates sonorously and is accompanied with seizures, rapid neurologic decline, vomiting, and different grades of liver disorder (3). No specific treatment or preventive method has been suggested for this disease, and poor prognosis with less than 10% chance of complete recovery is generally expected in these patients. Most of these patients experience rapid neurologic decline and death. The intensity of involvement and lesions on magnetic resonance imaging (MRI) are clearly related to the outcome of the disease (7). 

**Case 1**

A five-year-old boy was referred to Bu-Ali Hospital of Ardabil, Iran with fever (38.9°C) and vomiting. His level of consciousness decreased during admission, and repetitive seizures occurred on the second day of admission. He had a history of flu-like symptoms in the past week, but he was healthy otherwise and had a normal development. In the pediatric intensive care unit (PICU), the clinical examination showed light coma and responsiveness to pain. The pupils were miotic and non-responsive. Other cranial nerves were normal, and deep tendon reflexes (DTRs) were exaggerated. The Babinski reflex was positive, and the gag reflex was intact. His parents were cousins and had another child with sensorineural hearing loss and neurodevelopmental delay. 

The laboratory tests indicated the following results: white blood cell count (WBC)= 8900/μL (PMN: 45%; lymph: 50%; Eo: 2%; Mono: 3%); hemoglobin (Hb)= 12 g/dlit; hematocrit (Hc)= 35.5%; platelet (PLT)= 310,000/mm^3^; prothrombin time (PT)= 13 s; international normalized ratio (INR)= 1.1; partial thromboplastin time (PTT)= 31 s; blood sugar (BS)= 98 mg/dlit; blood urea nitrogen (BUN)= 30 mg/dlit; creatinine (Crt)= 1 mg/dlit; Na= 142 meq/lit; K= 4 meq/lit; alanine aminotransferase (ALT)= 218 IU/Lit; and aspartate aminotransferase (AST)= 139 IU/Lit. Serum ammonia, lactate, urinary organic acids, and metabolic screening were normal. 

High-performance liquid chromatography (HPLC) for the serum amino acid profile was normal. The cerebrospinal fluid (CSF) analysis indicated the following results: WBC=0; red blood cell count (RBC)=0; protein=12 mg/dlit; and BS=57 mg/dlit. The patient’s opening pressure was found to increase (30 Cm H_2_O). The results of PCR for enterovirus 71, parvovirus B19, Epstein–Barr virus (EBV), HSV, and influenza virus A and B were negative. Brain MRI showed abnormal signals and enlargement of both thalami, in addition to multiple bilateral T2W hyperintensities in the centrum semiovale and periventricular white matter, suggestive of ANE (Figure 1). 

Intravenous immunoglobulin (IVIG; 2 g/kg) and methylprednisolone (30 mg/kg/daily for five days) were initiated for treatment, and phenytoin was prescribed considering the patient’s consciousness level. After one week, his level of consciousness improved, whereas speech and motor disabilities persisted. He was discharged from the hospital and referred to an ergo-therapist for rehabilitation.

**Case 2:**

A three-year-old girl was referred to our hospital with restlessness, agitation, alteration of consciousness, and hallucinations. She had episodes of vomiting, diarrhea, and fever over the past 10 days. She was the child of consanguineous parents, and her brother had died due to encephalitis. She had one episode of seizure and was admitted to the PICU. In the physical examination, she showed loss of consciousness, disorientation, and lack of speech after improvement of alertness. Her pupils were normal, and the gag reflex and cranial nerves were intact.

The laboratory tests showed that WBC count, WBC differential, erythrocyte sedimentation rate (ESR), coagulation screening test, biochemistry tests, and metabolic screening were normal. ALT and AST were both normal, while alkaline phosphatase (ALP) had mildly increased, compatible with age. Arterial blood gas (ABG), lactate, ammonia, HPLC for serum amino acids, and urinary organoacids were normal. In the CSF analysis, all parameters were also normal (WBC, RBC, protein, glucose, and pressure). PCR for HSV was negative. Brain MRI showed multiple white matter lesions in the periventricular zone and left cerebellum, as well as hemorrhagic lesions in the pons with mass effect, without gadolinium ring enhancement and bilateral thalamus involvement (both T1W and T2W hyperintensity) (Figure 2).

For the purpose of treatment, methylprednisolone (30 mg/kg) and IVIG (2 g/kg) were prescribed for five days, which improved the patient’s level of consciousness. Her motor activities also progressed, and she could sit without support.

**Case 3:**

A four-year-old girl was referred to our hospital with a diagnosis of febrile convulsion. She had experienced episodes of vomiting after a febrile illness five days before admission. Respiratory and gastrointestinal symptoms were reported over the past week. Due to prolonged postictal phase (>48 hours), she was transferred to the PICU. Upon admission, she had some choreoathetoid movements, which disappeared with sleep. In the physical examination, she was in a state of stupor in her first visit. Her pupils were normal in size and reflex, but she had right sixth nerve palsy. DTRs were exaggerated, and extensor plantar reflexes were observed bilaterally. Her parents were not related, and there was no family history of neurologic disorders, except for multiple sclerosis in the father’s cousin.

The laboratory tests indicated the following results. Complete blood cell count (CBC), metabolic screening, electrolytes, and biochemistry results were normal, except for ALT and AST (135 and 138 IU/lit, respectively). The ALP level was measured to be 860 IU/lit. The CSF analysis revealed an increase in the opening pressure (40 Cm H_2_O), without any changes in WBC or RBC. The CSF glucose and protein levels were also normal. The results of PCR for enterovirus 71, influenza A and B, HSV, and parvovirus B19 were negative. Moreover, autoimmune encephalitis antibodies, including anti-Hu and anti-voltage-gated potassium channel (anti-VGKC) were evaluated, all of which were found to be negative. The brain MRI indicated lesions in the pons, as well as bilateral thalamus lesions and enlargement.

After prescribing five doses of methylprednisolone (30 mg/kg/daily) and 2 g/kg of IVIG, the patient’s general condition, speech, and motor function improved, and she was discharged from the hospital in a good condition.

## Discussion

ANEC or ANE refers to a rare and acute disease, which occurs after a respiratory or gastrointestinal viral infection with influenza virus, herpes virus, EBV, parvovirus b19, or enterovirus 71 in a previously healthy child. Although the most likely causes of ANEC are immune-mediated or metabolic, it has been reported that cytokines, such as TNF receptor 1, interleukin-1 (IL-1), and IL-6 can mediate the disease.

Mutations in the nuclear pore complex, RANBP2, contribute to familial and recurrent ANEC. Following the prodromal phase, patients experience episodes of seizure, vomiting, and alteration of consciousness. The elevated level of liver enzymes is seen in 70-80% of patients. In brain imaging, bilateral thalamotegmental involvement and necrosis of glial neurons and cerebellar dentate nucleus can be observed. Thalamic lesions may be hemorrhagic with lateral putamen involvement.

Differential diagnoses of ANEC include Reye syndrome, acute disseminated encephalomyelitis (ADEM), hemorrhagic shock syndrome, Japanese encephalitis, and inborn errors of metabolism. We ruled out the most common differential diagnosis (inborn errors of metabolism) by metabolic screening tests (serum amino acid HPLC, carnitine profile, urinary organic acids, lactate, and ammonia). In addition, the normal CSF (absence of pleocytosis and normal glucose) ruled out the diagnosis of encephalitis and ADEM. Similarly, ammonia was normal in our patients, which ruled out the diagnosis of Reye syndrome.

**In Conclusion**

Considering the family history of neurodevelopmental delay and unjustified death of siblings in cases 1 and 2, it may be necessary to check the patient’s history, such as metabolic and genetic disorders, to evaluate the occurrence of ANEC. It is worth mentioning that the co-occurrence of three cases of a rare disease in a short period may indicate the possibility of an epidemic. Since concurrent administration of methylprednisolone and IVIG resulted in good improvements, it is recommended to prescribe these drugs at the same time.

**Case 1:**
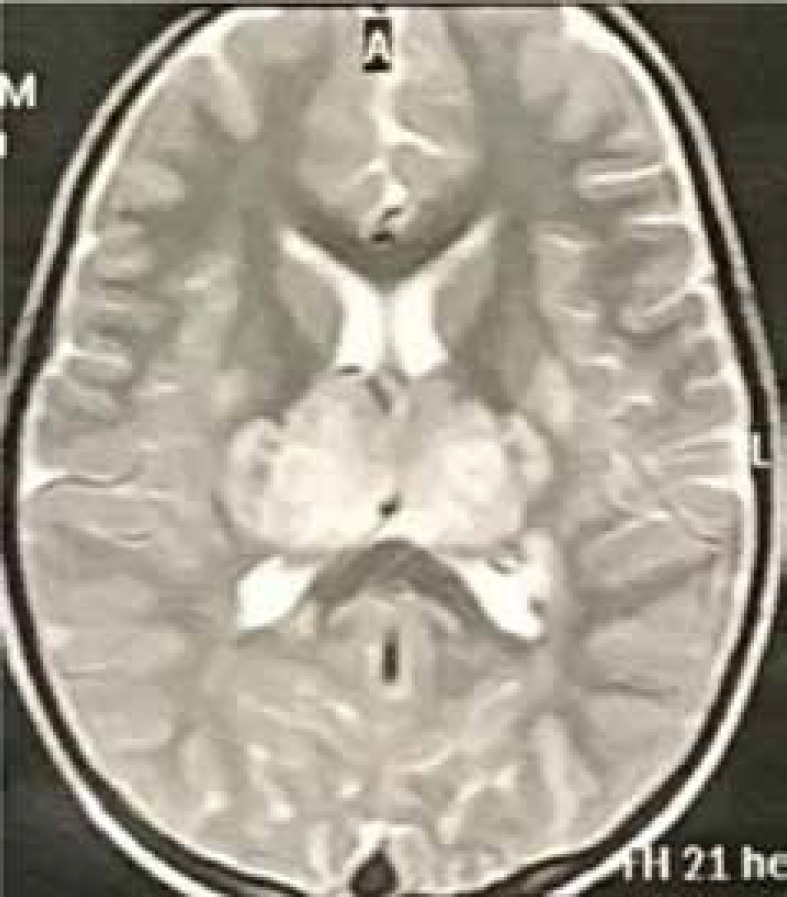
Bithalamic involvement in acute necrotizing encephalopathy (ANEC)

**Case 2:**
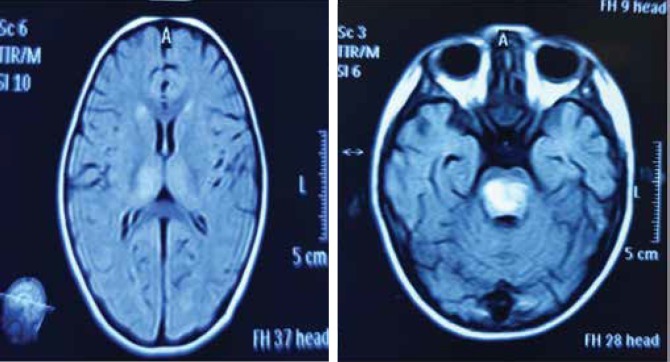
Thalamus and pons hyperintensities in the FLAIR sequence of ANEC

**Case 3:**
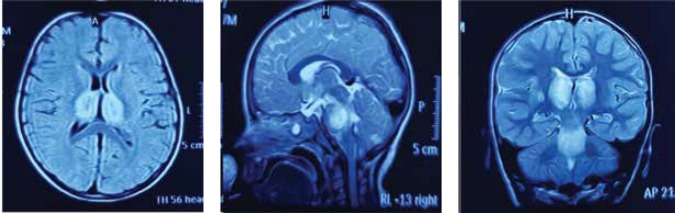
Bithalamic hypersignal lesions in the axial, sagittal, and coronal views
